# Genetic Analysis and QTL Detection for Resistance to White Tip Disease in Rice

**DOI:** 10.1371/journal.pone.0106099

**Published:** 2014-08-27

**Authors:** Tong Zhou, Cunyi Gao, Linlin Du, Hui Feng, Lijiao Wang, Ying Lan, Feng Sun, Lihui Wei, Yongjian Fan, Wenbiao Shen, Yijun Zhou

**Affiliations:** 1 Institute of Plant Protection, Jiangsu Academy of Agricultural Sciences, Nanjing, China; 2 College of Life Science, Nanjing Agricultural University, Nanjing, China; Institute of Botany, Chinese Academy of Sciences, China

## Abstract

The inheritance of resistance to white tip disease (WTDR) in rice (*Oryza sativa* L.) was analyzed with an artificial inoculation test in a segregating population derived from the cross between Tetep, a highly resistant variety that was identified in a previous study, and a susceptible cultivar. Three resistance-associated traits, including the number of *Aphelenchoides besseyi* (*A. besseyi*) individuals in 100 grains (NA), the loss rate of panicle weight (LRPW) and the loss rate of the total grains per panicle (LRGPP) were analyzed for the detection of the quantitative trait locus (QTL) in the population after construction of a genetic map. Six QTLs distributed on chromosomes 3, 5 and 9 were mapped. *qNA3* and *qNA9*, conferring reproduction number of *A. besseyi* in the panicle, accounted for 16.91% and 12.54% of the total phenotypic variance, respectively. *qDRPW5a* and *qDRPW5b*, associated with yield loss, were located at two adjacent marker intervals on chromosome 5 and explained 14.15% and 14.59% of the total phenotypic variation and possessed LOD values of 3.40 and 3.39, respectively. *qDRPW9* was considered as a minor QTL and only explained 1.02% of the phenotypic variation. *qLRGPP5* contributed to the loss in the number of grains and explained 10.91% of the phenotypic variation. This study provides useful information for the breeding of resistant cultivars against white tip disease in rice.

## Introduction

White tip disease of rice (WTDR), which is caused by the rice white tip nematode (*Aphelenchoides besseyi*, *A. besseyi*), is one of the most serious nematode diseases affecting rice worldwide [1]–[5]. Plants infected with *A. besseyi* exhibit whitening and withering at the tip of the leaf, and the symptoms also include small grains and erect panicles in later growth stages, which can result in large losses in production. The traditional methods used to control *A. besseyi* (including insecticide application and crop rotation) are expensive and cause serious environmental problems [4]. In addition, the wide-scale use of direct-sowing technology for rice, which is not compatible with the treatment of seeds, has recently made this problem more acute in China. Thus, the development of resistant rice cultivars has been considered as the primary strategy for controlling white tip disease [6], [7].

Rice varieties that are resistant against WTDR have been reported by researchers, e.g., *cvs Arkansas Fortuna*, *Nira 43*, *Asa-Hi*, *Binam* and *Domsiah*, which were screened from a large number of rice varieties [4], [8], [9]. However, the genetic mechanism of WTDR resistance is still poorly understood. In addition, some of the resistant varieties exhibited resistance to WTDR only in particular regions or were highly susceptible to other pathogens [10], which hindered projects aimed at breeding for resistance against WTDR. In addition, many nematode resistance loci, such as *H1*, *GroV1*, *Cre* and *Mi3*, have been identified in tomato, potato, soybean and other crops [11]–[14], and some have even been cloned and functionally analyzed [15]–[18]. In addition, a gene (*Has-1^Og^*) resistant to the cyst nematode (*Heterodera sacchari*) has been identified in rice [19]. These results suggested that host plants, including rice, harbored defense mechanisms to fight against nematodes, and these mechanisms have developed over long-term co-evolution. The lack of information on the inheritance of the resistance to WTDR may slow the progress of breeding programs. Therefore, elucidating the resistance mechanisms involved will contribute to a better understanding of nematode-plant interactions and assist with the breeding of nematode-resistant cultivars [20].

In our previous study [21], a collection of germplasm resources was screened using an inoculation test, and an *indica* variety, Tetep, showed high resistance to WTDR. Herein, we present the inheritance mode of resistance to WTDR in rice and the quantitative trait loci (QTLs) related to resistance against WTDR.

## Materials and Methods

### Plant materials

In 2008, Huaidao No.5, a *japonica* cultivar highly susceptible to WTDR, and Tetep (an *indica* variety) were grown at the experimental station in Jiangsu Academy of Agricultural Sciences. In the same year, F_1_ was developed from a cross between Huaidao No.5 and Tetep. In 2009, the F_1_ was grown and self-pollinated at the experimental station to generate F_2_ lines that were used as mapping populations in Nanjing, Jiangsu Province. In 2009–2010, the F_2_ lines were grown at the experimental station in Lingshui, Hainan Province, and the leaves of the F_2_ lines were collected, numbered and stored at −70°C. A total of 138 F_2_ individuals were selected and self-pollinated to generate 138 F_2∶3_ families. The resistance of the two parents, the F_1_ generation and the F_2∶3_ lines were evaluated at the experimental station in Jiangsu Academy of Agricultural Sciences in 2012. Seeds were pretreated by soaking them in water at 55°C for 15 minutes to ensure there were no live *A. besseyi* before the seeds were sown [4].

### Nematode preparation

The seed-borne ectoparasitic *A. besseyi* was initially isolated from infected rice seeds using the Baermann funnel technique and surface-sterilized with 3% H_2_O_2_ for 10 minutes. After isolation, *A. besseyi* was cultured on *Botrytis cinerea*, which were grown on potato dextrose agar (PDA) medium for approximately 3 weeks at 25°C [22]. Then, the nematodes were rinsed thoroughly with distilled water and used as inoculation material [23].

### Evaluation of resistance

40-µl nematode suspension with 400 juveniles of *A. besseyi* was inoculated between the leaf sheath and the culm with a pipette at the top tillering stage. To keep the micro-environment moist so that the nematodes were able to move and feed easily, a small wad of absorbent cotton was placed in the infection spot [7], [21], [24]. The mature seeds of each inoculated panicle were harvested. The number of grains per panicle and the weight of the panicle were determined. The number of nematodes in 100 grains from each inoculated panicle was counted using the following protocol. The peeled seed and the shell were soaked in distilled water for 24 hours, after which the free *A. besseyi* were collected from the solution using the Baermann funnel technique, and a microscope was used to count the number of *A. besseyi* [21]. At least 2 panicles of 25 plants from each F_2∶3_ line were used for the inoculation. Distilled water without *A. besseyi* was used as the control.

The number of *A. besseyi* individuals in 100 grains (NA), the loss rate of panicle weight (LRPW) and the loss rate of the total grains per panicle (LRGPP) were calculated using the following formulas: NA = (the total number of *A. besseyi* in the counted grains/the total number of counted grains)×100, LRPW = (the panicle weight of the control–the panicle weight of the inoculated plant)/the panicle weight of the control and LRGPP = (the total number of grains per panicle in the control–the total number of grains per panicle in the inoculated plant)/the total number of grains per panicle in the control.

### Genotyping, linkage map construction and QTL analysis

The DNA of the two parents, the F_1_ generation and the 138 F_2_ lines was extracted using the CTAB method described by Rogers and Bendich [25]. SSR markers (842 pairs), obtained from Gramene (http://www.gramene.org), and polymorphism markers for the two parents were tested. PCR amplification was performed as described by Septiningsih et al. [26], and PCR products were detected using 8% denaturing polyacrylamide gel electrophoresis. The polymorphic markers were used for genotype analysis of the F_2_ lines to assemble the linkage map. The genetic linkage map was constructed by employing MAPMAKER/EXP 3.0 software [27], [28]. Marker distances in centimorgans (cM) were calculated using the Kosambi function.

Composite interval mapping (CIM) was applied to analyze the phenotypic and genotypic data for detecting the QTLs responsible for resistance to *A. besseyi* by using Windows QTL Cartographer 2.5 software [29]. Experiment-wide significance (P<0.05) threshold values of the LOD scores for putative QTL detection were determined with 1000 permutations. The threshold value of the LOD was 2.5 at a significance level of P = 0.05 for CIM. The additive effect and the explanation of the phenotypic variance for each QTL were also acquired using this software.

## Results

### Phenotypic variation

**Figure 1:**
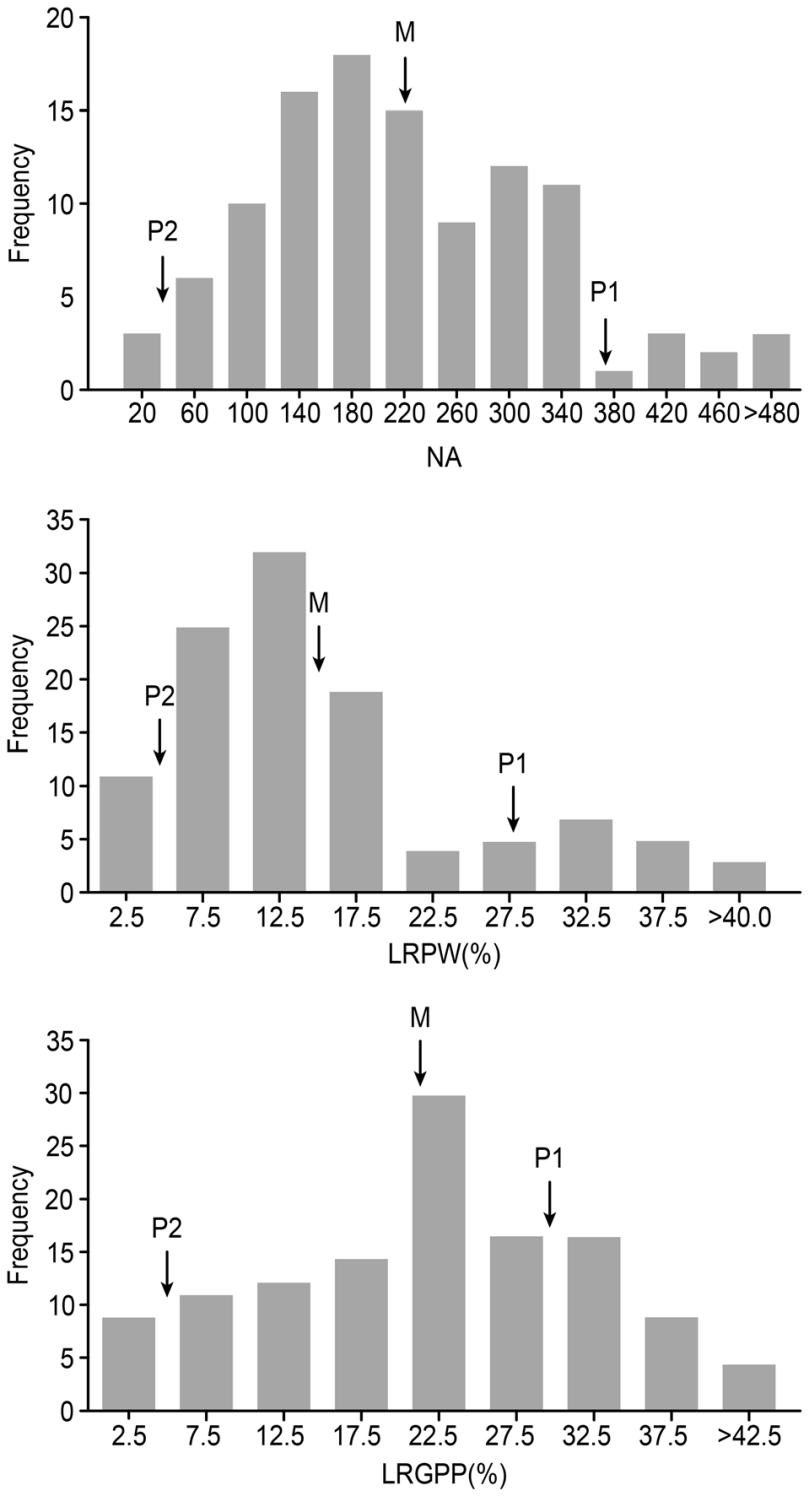
The frequency distributions of three resistance-associated traits in the F_2∶3_ lines. P_1_ represents Huaidao No.5, P_2_ represents Tetep, and M represents the mean value of the F_2∶3_ lines.

A dramatic difference in NA, LRPW and LRGPP was observed between Huaidao No.5 and Tetep (Table 1). The values for NA, LRPW and LRGPP of Huaidao No.5 were 472, 0.28 and 0.29, respectively. In comparison, the values for Tetep were much lower, at 51, 0.04 and 0.05, respectively. These data supported the hypothesis that Huaidao No.5 was highly susceptible to WTDR, whereas Tetep was highly resistant. The values for NA, LRPW and LRGPP of the F_2∶3_ lines ranged from 24 to 514, from 0.01 to 0.44 and from 0 to 0.64, respectively. Each of the frequency distributions for NA, LRPW and LRGPP showed a continuous distribution, which indicated that these traits behaved as quantitative variables (Figure 1).

**Table 1:** Three performance traits of the two parents and the F_2∶3_ lines.

Traits	Huaidao No.5	Tetep	|P_1_–P_2_|	F_2∶3_ lines	
				Mean±SD	Range
NA	472±80	51±19	421	222±110	24–514
LRPW	0.28±0.19	0.04±0.01	0.24	0.15±0.10	0.01–0.44
LRGPP	0.29±0.09	0.05±0.01	0.24	0.22±0.13	0.00–0.64

P_1_ and P_2_ represent Huaidao No.5 and Tetep, respectively.

### Correlation coefficients among the three resistance-associated traits

The correlation analysis among the three resistance-associated traits was performed using SPSS ver. 20.0 software (Table 2). The results showed that NA was not significantly correlated with LRPW or LRGPP. However, a significant positive correlation was observed between LRPW and LRGPP at the P<0.01 level.

**Table 2:** Correlation coefficients among the three resistance-associated traits.

Traits	NA	LRPW	LRGPP
NA	1		
LRPW	0.022	1	
LRGPP	−0.059	0.424**	1

**Significant at the 0.01 level.

### Genetic linkage map

A total of 160 polymorphic SSR markers were found between the two parents of 842 total markers, for a ratio of 19.01%. A linkage map was constructed, which included 12 linkage groups and spanned a total of 2179.6 cM in genetic distance with an average of 17.16 cM among 127 SSR polymorphic markers. Because these SSR markers were evenly distributed on 12 chromosomes, the linkage map was suitable for QTL detection.

### QTL analysis

**Figure 2:**
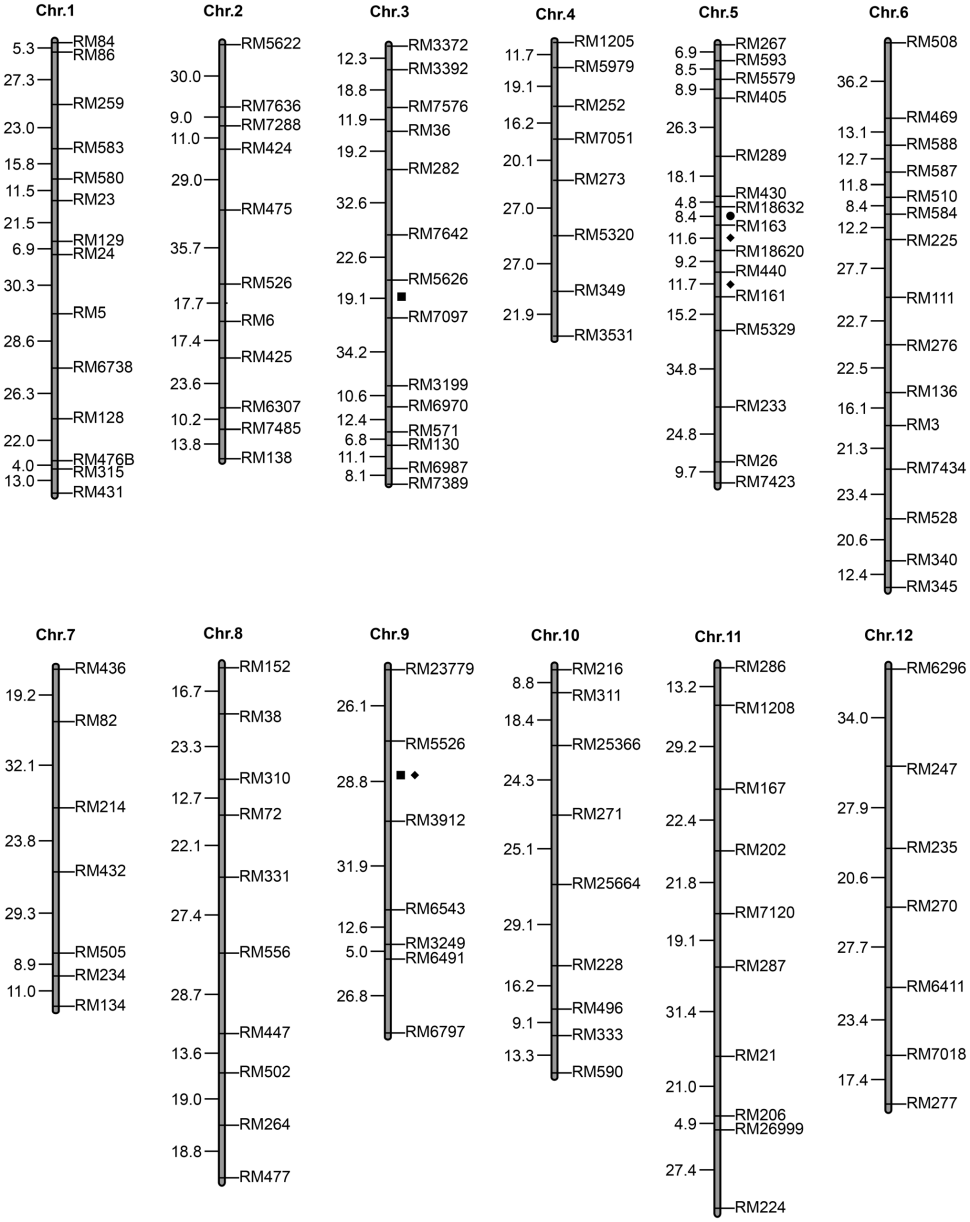
The SSR linkage map and chromosomal distributions of the putative loci responsible for resistance to WTDR. ▪, ⧫ and • indicate the resistance QTLs detected using the resistance-associated traits of NA, LRPW and LRGPP, respectively.

QTL identification using CIM indicated that a total of six QTLs for resistance to WTDR were found on chromosomes 3, 5 and 9 (Table 3, Figure 2). Two important QTLs (*qNA3* and *qNA9*) responsible for the reproduction numbers of *A. bessey*i in the panicles were detected at the marker intervals of RM5626–RM7097 and RM5526–RM3912 on chromosomes 3 and 9, respectively. The LOD values of *qNA3* and *qNA9* were 3.04 and 2.62, which accounted for 16.91% and 12.54% of the total phenotypic variation, respectively. Three QTLs, i.e., *qLRPW5a*, *qLRPW5b* and *qLRPW9*, which conferred yield loss, were mapped at the marker intervals of RM163–RM18620, RM440–RM161 and RM5526–RM3912, respectively, on chromosomes 5 and 9 (Table 3). *qLRPW5a* and *qLRPW5b*, which were located at two adjacent marker intervals on chromosome 5, explained 14.15% and 14.59% of the phenotypic variation and possessed LOD values 3.40 and 3.39, respectively. *qLRPW9* was considered as a minor QTL because it only explained 1.02% of the phenotypic variation and had an LOD value of 2.55. We also identified a QTL (*qLRGPP5*) responsible for the loss in the number of the grains at the marker interval RM18632–RM163 on chromosome 5 (Table 3), which explained 10.91% of the phenotypic variation. Among these QTLs, four (*qNA3*, *qLRPW5a*, *qLRPW5b* and *qLRGPP5*) were derived from Tetep, and the others (*qNA9* and *qLRPW9*) originated from Huaidao No.5.

**Table 3:** QTLs for resistance to WTDR detected in the F_2_ lines developed from crossing Huaidao No.5 and Tetep.

Traits	QTL	Chr.	Interval	R^2^ (%)	A (%)	LOD
NA	*qNA3*	3	RM5626-RM7097	16.91	71.18	3.04
	*qNA9*	9	RM5526-RM3912	12.54	−61.08	2.62
LRPW	*qLRPW5a*	5	RM163-RM18620	14.15	5.43	3.40
	*qLRPW5b*	5	RM440-RM161	14.59	5.51	3.39
	*qLRPW9*	9	RM5526-RM3912	1.02	−1.5	2.55
LRGPP	*qLRGPP5*	5	RM18632-RM163	10.91	1.94	2.83

QTL represents the putative QTL at an LOD level ≥2.5.

Chr. represents Chromosome.

R^2^ represents the phenotypic variance of each QTL.

A is an additive effect.

## Discussion

It has been reported that not all the susceptible rice varieties exhibited symptoms of both white leaf tips and small grains after infection with *A. besseyi*, although plants without symptoms still showed large losses in production [21]. Therefore, the relative yield loss was preferentially used in this study to evaluate the resistance level of plants against WTDR, which was described by the LRPW. The final number of nematodes in the mature grains was considered as an important assessment index by most researchers because of the potential transmission of the nematodes by seeds and because nematodes can contribute to the loss of the grain, which directly reduces the harvest. The number of *A. besseyi* in 100 grains was used as another assessment index based on a previous study [21]. We also used the LRGPP to measure the loss in the number of grains, which caused the small grain symptoms. Another symptom, i.e., white leaf tips, was barely seen in our study, which was reported by Feng [21] and Fortuner [30], and was not investigated. Correlation analysis showed that there was no relationship between NA and LRPW or LRGPP. This finding suggested that the reproduction level of *A. besseyi* in the grains was not entirely responsible for the loss in production and that there may be unknown factors involved in the resistance process. Roberts [31] believed there was tolerance factor involved in reducing the yield loss caused by WTDR. In contrast, LRPW was significantly correlated with LRGPP, which indicated that the decrease in the number of grains in the mature panicles infected by *A. besseyi* caused a direct loss in yield. The mapping results confirmed this hypothesis, i.e., one of the loci for LRPW, *qLRPW5a*, was close to *qLRGPP5*, a locus for LRGPP. *qLRPW9* and *qNA9* were close to each other, but there was no relationship between these two traits. This lack of a relationship might stem from the fact that *qLRPW9* was a minor QTL and only accounted for 1.02% the phenotypic variation.

In this study, we identified 6 QTLs related to resistance against WTDR. Three important QTLs (*qLRPW5a*, *qLRPW5b* and *qLRGPP5*) located on chromosome 5 contributed to a decrease in the yield loss and grain number loss caused by WTDR. There were several genes related to the number, yield and weight of the grain, including *gw-5*, *gw5-1*, *Gwt5a*, *tgwt5* and *qYI-5* in the region of *qLRPW5a* and *qLRGPP5*, which were very close to each other [32]–[36]. The region containing another QTL (*qLRPW5b*) also included yield-related genes such as *gp5*, *QGwp5*, *gwt5*, *Pdw5*, *ssp-5* and *tgwt5b* [37]–[41]. This pattern suggests that these genes may be involved in reducing the damage in the grains after infection by *A. besseyi*, i.e., although these genes do not contribute directly to resistance against WTDR, they are involved in yield protection and are useful for resistance breeding projects. The resistance loci responsible for different types of pathogens are thought to be clustered in a chromosomal region in many organisms [42]. We found several resistance genes (e.g., *qBB3-1* and *rbr3*) against pathogens (rice bacterial blight and rice blast) by comparative mapping using a common marker [43], [44]. Additionally, the additive effect of *qNA9* and *qLRPW9* were −61.8% and −1.5%, respectively, i.e. the resistance of these two QTLs originated from Huaidao No.5. It suggested that Huaidao No.5, a susceptible variety, also possessed the resistance loci. Lorieux et al. [19] mapped a resistance gene (*Has-1^Og^*) to the cyst nematode on rice chromosome 11 between two markers, i.e., RM206 and RM254. In our study, however, no gene was identified on chromosome 11, most likely because different resistance mechanisms occurring in rice depended on the different types of nematodes.

Recently, the *A. besseyi* infestation has caused increasing losses in rice yield in China [21], [45]–[47]. Breeding and utilizing resistant cultivars is considered the most effective strategy for resolving this urgent problem. In this study, we identified the QTLs responsible for resistance to WTDR, which can be further improved by fine mapping to identify the tightly linked markers. Based on these findings, molecular-level breeding efforts target at WTDR can be more efficient, especially for locating both the nematode-resistance genes and the yield-protection genes.

It was reported previously that some resistance genes against nematodes had been cloned. Functional analysis has shown that these resistance genes can be divided into two types. The first type, which possessed a nucleotide binding site-leucine-rich repeat (NBS-LRR) structure, was located in the cytoplasm but lacked a signal sequence, e.g., *Mi-1*, *HeroA*, *Gpa2* and *Gro1-4* [15], [17], [18], [20]. The second type has a transmembrane structure and a signal sequence, e.g., *Hs1^pro-1^*, *Rhg1*, and *Rhg4* [16], [48]. Hosts have evolved various defense pathways to protect themselves against invading nematode diseases. The vast amount of available information can help to rapidly and easily identify the set of resistance genes in this cultivar.
